# Supplementary material for: Learning to detect AI texts and learning the limits
Source: PLoS One. 2025 Oct 15;20(10):e0333007. doi: 10.1371/journal.pone.0333007 (PMC12527182; doi:10.1371/journal.pone.0333007)
Supplement: S2 File — All data and scripts used in the study, some additional descriptive statistics, scalable formate figures. (ZIP) [file pone.0333007.s002.tgz › data and scripts/OSF/scripts/analysis/mixed models jamovi/outputs/complete_correctness_nonlearners.html]

Results


# Generalized Mixed Model

| Model Info | | | | | |
| --- | --- | --- | --- | --- | --- |
| Info | | Value | | Comment | |
| Model Type |  | Logistic |  | Model for binary y |  |
| Call |  | glm |  | Is correct ~ 1 + More readable is human + Confidence + Demographics: age + Demographics: education\_ordinal + Demographics: ai\_usage\_ordinal + Demographics: gender merged + Demographics: field\_merged + Reaction time log + GLS Euclidean distance + GLS1 difference + GLS2 difference + GLS3 difference + GLS4 difference + id + Demographics: reading\_disorder\_merged + GLS8 difference + GLS7 difference + GLS6 difference + GLS5 difference + Demographics: ai\_improve + Demographics: ai\_risk + Demographics: ai\_quality + Demographics: ai\_lacks\_creativity + Demographics: ai\_lacks\_understanding + Demographics: ai\_grammar + Demographics: ai\_avoidance + Demographics: ai\_dumbing + Demographics: ai\_fascination + (0 + More readable is human + Confidence + Reaction time log + GLS Euclidean distance + GLS1 difference + GLS2 difference + GLS3 difference + GLS4 difference + id + GLS8 difference + GLS7 difference + GLS6 difference + GLS5 difference \| Participant ID) |  |
| Link function |  | Logit |  | Log of the odd of y=1 over y=0 |  |
| Direction |  | P(y=1)/P(y=0) |  | P( Is correct = 1 ) / P( Is correct = 0 ) |  |
| Distribution |  | Binomial |  | Dichotomous event distribution of y |  |
| LogLikel. |  | -1287.871 |  | Unconditional Log-Likelihood |  |
| -2\*LogLikel. |  | 2575.743 |  | Unconditional absolute deviance |  |
| Deviance |  | 1994.258 |  | Conditional relative deviance |  |
| R-squared |  | NaN |  | Marginal |  |
| R-squared |  | NaN |  | Conditional |  |
| AIC |  | 2851.740 |  | Less is better |  |
| BIC |  | 3640.553 |  | Less is better |  |
| Residual DF |  | 2106.000 |  |  |  |
| Chi-squared/DF |  | 0.738 |  | Overdispersion indicator |  |
| Converged |  | yes |  |  |  |
| Optimizer |  | bobyqa |  |  |  |
|  |  |  |  |  |  |
| --- | --- | --- | --- | --- | --- |
| Note. R-squared cannot be computed. | | | | | |
| Note. boundary (singular) fit: see ?isSingular | | | | | |
|  | | | | | |

## Model Results

| Fixed Effect Omnibus tests | | | | | | | |
| --- | --- | --- | --- | --- | --- | --- | --- |
|  | | X² | | df | | p | |
| More readable is human |  | 89.7178 |  | 1.00 |  | < .001 |  |
| Confidence |  | 8.0146 |  | 1.00 |  | 0.005 |  |
| Demographics: age |  | 1.8030 |  | 1.00 |  | 0.179 |  |
| Demographics: education\_ordinal |  | 0.4735 |  | 1.00 |  | 0.491 |  |
| Demographics: ai\_usage\_ordinal |  | 0.0771 |  | 1.00 |  | 0.781 |  |
| Demographics: gender merged |  | 0.7445 |  | 2.00 |  | 0.689 |  |
| Demographics: field\_merged |  | 1.7027 |  | 4.00 |  | 0.790 |  |
| Reaction time log |  | 1.1086 |  | 1.00 |  | 0.292 |  |
| GLS Euclidean distance |  | 3.7821 |  | 1.00 |  | 0.052 |  |
| GLS1 difference |  | 12.4916 |  | 1.00 |  | < .001 |  |
| GLS2 difference |  | 6.2612 |  | 1.00 |  | 0.012 |  |
| GLS3 difference |  | 5.0625 |  | 1.00 |  | 0.024 |  |
| GLS4 difference |  | 5.7322 |  | 1.00 |  | 0.017 |  |
| id |  | 1.1919 |  | 1.00 |  | 0.275 |  |
| Demographics: reading\_disorder\_merged |  | 1.0745 |  | 1.00 |  | 0.300 |  |
| GLS8 difference |  | 1.4838 |  | 1.00 |  | 0.223 |  |
| GLS7 difference |  | 0.6111 |  | 1.00 |  | 0.434 |  |
| GLS6 difference |  | 0.0648 |  | 1.00 |  | 0.799 |  |
| GLS5 difference |  | 2.5921 |  | 1.00 |  | 0.107 |  |
| Demographics: ai\_improve |  | 1.2544 |  | 1.00 |  | 0.263 |  |
| Demographics: ai\_risk |  | 0.1927 |  | 1.00 |  | 0.661 |  |
| Demographics: ai\_quality |  | 0.0296 |  | 1.00 |  | 0.863 |  |
| Demographics: ai\_lacks\_creativity |  | 0.0253 |  | 1.00 |  | 0.874 |  |
| Demographics: ai\_lacks\_understanding |  | 1.2504 |  | 1.00 |  | 0.263 |  |
| Demographics: ai\_grammar |  | 12.8382 |  | 1.00 |  | < .001 |  |
| Demographics: ai\_avoidance |  | 1.7876 |  | 1.00 |  | 0.181 |  |
| Demographics: ai\_dumbing |  | 1.0169 |  | 1.00 |  | 0.313 |  |
| Demographics: ai\_fascination |  | 0.0266 |  | 1.00 |  | 0.871 |  |
|  |  |  |  |  |  |  |  |
| --- | --- | --- | --- | --- | --- | --- | --- |
|  | | | | | | | |

| Fixed Effects Parameter Estimates | | | | | | | | | | | | | | | | | | | | | |
| --- | --- | --- | --- | --- | --- | --- | --- | --- | --- | --- | --- | --- | --- | --- | --- | --- | --- | --- | --- | --- | --- |
|  | | | | | | | | 95% Confidence Interval | | | |  | | 95% Exp(B) Confidence Interval | | | |  | | | |
| Names | | Effect | | Estimate | | SE | | Lower | | Upper | | exp(B) | | Lower | | Upper | | z | | p | |
| (Intercept) |  | (Intercept) |  | 1.07430 |  | 0.3503 |  | 0.38767 |  | 1.76092 |  | 2.928 |  | 1.474 |  | 5.818 |  | 3.0666 |  | 0.002 |  |
| More readable is human1 |  | 1 - 0 |  | 2.06307 |  | 0.2178 |  | 1.63617 |  | 2.48996 |  | 7.870 |  | 5.135 |  | 12.061 |  | 9.4719 |  | < .001 |  |
| Confidence |  | Confidence |  | -0.13657 |  | 0.0482 |  | -0.23113 |  | -0.04202 |  | 0.872 |  | 0.794 |  | 0.959 |  | -2.8310 |  | 0.005 |  |
| Demographics: age |  | Demographics: age |  | 0.01868 |  | 0.0139 |  | -0.00859 |  | 0.04594 |  | 1.019 |  | 0.991 |  | 1.047 |  | 1.3428 |  | 0.179 |  |
| Demographics: education\_ordinal |  | Demographics: education\_ordinal |  | 0.08890 |  | 0.1292 |  | -0.16431 |  | 0.34210 |  | 1.093 |  | 0.848 |  | 1.408 |  | 0.6881 |  | 0.491 |  |
| Demographics: ai\_usage\_ordinal |  | Demographics: ai\_usage\_ordinal |  | 0.01728 |  | 0.0623 |  | -0.10473 |  | 0.13929 |  | 1.017 |  | 0.901 |  | 1.149 |  | 0.2777 |  | 0.781 |  |
| Demographics: gender merged1 |  | male - female |  | -0.01249 |  | 0.1846 |  | -0.37436 |  | 0.34938 |  | 0.988 |  | 0.688 |  | 1.418 |  | -0.0676 |  | 0.946 |  |
| Demographics: gender merged2 |  | other - female |  | -0.44541 |  | 0.5175 |  | -1.45971 |  | 0.56890 |  | 0.641 |  | 0.232 |  | 1.766 |  | -0.8607 |  | 0.389 |  |
| Demographics: field\_merged1 |  | human - cs |  | -0.38585 |  | 0.3722 |  | -1.11542 |  | 0.34372 |  | 0.680 |  | 0.328 |  | 1.410 |  | -1.0366 |  | 0.300 |  |
| Demographics: field\_merged2 |  | nature - cs |  | -0.29247 |  | 0.3946 |  | -1.06583 |  | 0.48089 |  | 0.746 |  | 0.344 |  | 1.618 |  | -0.7412 |  | 0.459 |  |
| Demographics: field\_merged3 |  | other - cs |  | -0.36696 |  | 0.4746 |  | -1.29717 |  | 0.56326 |  | 0.693 |  | 0.273 |  | 1.756 |  | -0.7732 |  | 0.439 |  |
| Demographics: field\_merged4 |  | philology - cs |  | -0.51098 |  | 0.4184 |  | -1.33099 |  | 0.30904 |  | 0.600 |  | 0.264 |  | 1.362 |  | -1.2213 |  | 0.222 |  |
| Reaction time log |  | Reaction time log |  | -0.08757 |  | 0.0832 |  | -0.25057 |  | 0.07544 |  | 0.916 |  | 0.778 |  | 1.078 |  | -1.0529 |  | 0.292 |  |
| GLS Euclidean distance |  | GLS Euclidean distance |  | 0.09811 |  | 0.0504 |  | -7.66e−4 |  | 0.19699 |  | 1.103 |  | 0.999 |  | 1.218 |  | 1.9448 |  | 0.052 |  |
| GLS1 difference |  | GLS1 difference |  | -0.30027 |  | 0.0850 |  | -0.46679 |  | -0.13376 |  | 0.741 |  | 0.627 |  | 0.875 |  | -3.5344 |  | < .001 |  |
| GLS2 difference |  | GLS2 difference |  | -0.33332 |  | 0.1332 |  | -0.59441 |  | -0.07224 |  | 0.717 |  | 0.552 |  | 0.930 |  | -2.5022 |  | 0.012 |  |
| GLS3 difference |  | GLS3 difference |  | 0.09330 |  | 0.0415 |  | 0.01203 |  | 0.17458 |  | 1.098 |  | 1.012 |  | 1.191 |  | 2.2500 |  | 0.024 |  |
| GLS4 difference |  | GLS4 difference |  | 0.11974 |  | 0.0500 |  | 0.02172 |  | 0.21776 |  | 1.127 |  | 1.022 |  | 1.243 |  | 2.3942 |  | 0.017 |  |
| id |  | id |  | -0.01233 |  | 0.0113 |  | -0.03447 |  | 0.00981 |  | 0.988 |  | 0.966 |  | 1.010 |  | -1.0917 |  | 0.275 |  |
| Demographics: reading\_disorder\_merged1 |  | true - false |  | 0.71990 |  | 0.6945 |  | -0.64126 |  | 2.08105 |  | 2.054 |  | 0.527 |  | 8.013 |  | 1.0366 |  | 0.300 |  |
| GLS8 difference |  | GLS8 difference |  | -0.04195 |  | 0.0344 |  | -0.10945 |  | 0.02555 |  | 0.959 |  | 0.896 |  | 1.026 |  | -1.2181 |  | 0.223 |  |
| GLS7 difference |  | GLS7 difference |  | 0.03550 |  | 0.0454 |  | -0.05350 |  | 0.12450 |  | 1.036 |  | 0.948 |  | 1.133 |  | 0.7817 |  | 0.434 |  |
| GLS6 difference |  | GLS6 difference |  | 0.00881 |  | 0.0346 |  | -0.05902 |  | 0.07664 |  | 1.009 |  | 0.943 |  | 1.080 |  | 0.2545 |  | 0.799 |  |
| GLS5 difference |  | GLS5 difference |  | -0.08746 |  | 0.0543 |  | -0.19392 |  | 0.01901 |  | 0.916 |  | 0.824 |  | 1.019 |  | -1.6100 |  | 0.107 |  |
| Demographics: ai\_improve |  | Demographics: ai\_improve |  | 0.08242 |  | 0.0736 |  | -0.06181 |  | 0.22666 |  | 1.086 |  | 0.940 |  | 1.254 |  | 1.1200 |  | 0.263 |  |
| Demographics: ai\_risk |  | Demographics: ai\_risk |  | -0.02545 |  | 0.0580 |  | -0.13910 |  | 0.08820 |  | 0.975 |  | 0.870 |  | 1.092 |  | -0.4389 |  | 0.661 |  |
| Demographics: ai\_quality |  | Demographics: ai\_quality |  | -0.01152 |  | 0.0670 |  | -0.14286 |  | 0.11981 |  | 0.989 |  | 0.867 |  | 1.127 |  | -0.1720 |  | 0.863 |  |
| Demographics: ai\_lacks\_creativity |  | Demographics: ai\_lacks\_creativity |  | -0.01039 |  | 0.0654 |  | -0.13853 |  | 0.11774 |  | 0.990 |  | 0.871 |  | 1.125 |  | -0.1590 |  | 0.874 |  |
| Demographics: ai\_lacks\_understanding |  | Demographics: ai\_lacks\_understanding |  | 0.07057 |  | 0.0631 |  | -0.05313 |  | 0.19427 |  | 1.073 |  | 0.948 |  | 1.214 |  | 1.1182 |  | 0.263 |  |
| Demographics: ai\_grammar |  | Demographics: ai\_grammar |  | 0.23112 |  | 0.0645 |  | 0.10469 |  | 0.35754 |  | 1.260 |  | 1.110 |  | 1.430 |  | 3.5830 |  | < .001 |  |
| Demographics: ai\_avoidance |  | Demographics: ai\_avoidance |  | -0.07768 |  | 0.0581 |  | -0.19155 |  | 0.03619 |  | 0.925 |  | 0.826 |  | 1.037 |  | -1.3370 |  | 0.181 |  |
| Demographics: ai\_dumbing |  | Demographics: ai\_dumbing |  | 0.05286 |  | 0.0524 |  | -0.04988 |  | 0.15559 |  | 1.054 |  | 0.951 |  | 1.168 |  | 1.0084 |  | 0.313 |  |
| Demographics: ai\_fascination |  | Demographics: ai\_fascination |  | -0.01330 |  | 0.0816 |  | -0.17320 |  | 0.14661 |  | 0.987 |  | 0.841 |  | 1.158 |  | -0.1630 |  | 0.871 |  |
|  |  |  |  |  |  |  |  |  |  |  |  |  |  |  |  |  |  |  |  |  |  |
| --- | --- | --- | --- | --- | --- | --- | --- | --- | --- | --- | --- | --- | --- | --- | --- | --- | --- | --- | --- | --- | --- |
|  | | | | | | | | | | | | | | | | | | | | | |

| Random Components | | | | | | | | | |
| --- | --- | --- | --- | --- | --- | --- | --- | --- | --- |
| Groups | | Name | | SD | | Variance | | ICC | |
| Participant ID |  | More readable is human0 |  | 0.9265 |  | 0.85845 |  |  |  |
|  |  | More readable is human1 |  | 1.0687 |  | 1.14205 |  |  |  |
|  |  | Confidence |  | 0.3027 |  | 0.09162 |  |  |  |
|  |  | Reaction time log |  | 0.1454 |  | 0.02113 |  |  |  |
|  |  | GLS Euclidean distance |  | 0.1909 |  | 0.03645 |  |  |  |
|  |  | GLS1 difference |  | 0.4111 |  | 0.16902 |  |  |  |
|  |  | GLS2 difference |  | 0.6810 |  | 0.46382 |  |  |  |
|  |  | GLS3 difference |  | 0.1320 |  | 0.01743 |  |  |  |
|  |  | GLS4 difference |  | 0.1857 |  | 0.03448 |  |  |  |
|  |  | id |  | 0.0494 |  | 0.00244 |  |  |  |
|  |  | GLS8 difference |  | 0.0338 |  | 0.00114 |  |  |  |
|  |  | GLS7 difference |  | 0.1193 |  | 0.01423 |  |  |  |
|  |  | GLS6 difference |  | 0.1179 |  | 0.01389 |  |  |  |
|  |  | GLS5 difference |  | 0.1486 |  | 0.02208 |  |  |  |
| Residuals |  |  |  | 1.0000 |  | 1.00000 |  | . |  |
|  |  |  |  |  |  |  |  |  |  |
| --- | --- | --- | --- | --- | --- | --- | --- | --- | --- |
| Note. Number of Obs: 2244 , groups: Participant ID 132 | | | | | | | | | |
|  | | | | | | | | | |

| Random Parameters correlations | | | | | | | |
| --- | --- | --- | --- | --- | --- | --- | --- |
| Groups | | Param.1 | | Param.2 | | Corr. | |
| Participant ID |  | More readable is human0 |  | More readable is human1 |  | -0.1357 |  |
|  |  | More readable is human0 |  | Confidence |  | 0.7366 |  |
|  |  | More readable is human0 |  | Reaction time log |  | -0.5014 |  |
|  |  | More readable is human0 |  | GLS Euclidean distance |  | -0.3042 |  |
|  |  | More readable is human0 |  | GLS1 difference |  | 0.4502 |  |
|  |  | More readable is human0 |  | GLS2 difference |  | -0.7730 |  |
|  |  | More readable is human0 |  | GLS3 difference |  | -0.1628 |  |
|  |  | More readable is human0 |  | GLS4 difference |  | 0.1273 |  |
|  |  | More readable is human0 |  | id |  | 0.8072 |  |
|  |  | More readable is human0 |  | GLS8 difference |  | 0.6546 |  |
|  |  | More readable is human0 |  | GLS7 difference |  | 0.5294 |  |
|  |  | More readable is human0 |  | GLS6 difference |  | 0.0181 |  |
|  |  | More readable is human0 |  | GLS5 difference |  | 0.1757 |  |
|  |  | More readable is human1 |  | Confidence |  | -0.6185 |  |
|  |  | More readable is human1 |  | Reaction time log |  | -0.1658 |  |
|  |  | More readable is human1 |  | GLS Euclidean distance |  | -0.2715 |  |
|  |  | More readable is human1 |  | GLS1 difference |  | -0.3666 |  |
|  |  | More readable is human1 |  | GLS2 difference |  | 0.1657 |  |
|  |  | More readable is human1 |  | GLS3 difference |  | 0.1708 |  |
|  |  | More readable is human1 |  | GLS4 difference |  | -0.2312 |  |
|  |  | More readable is human1 |  | id |  | -0.0489 |  |
|  |  | More readable is human1 |  | GLS8 difference |  | 0.0591 |  |
|  |  | More readable is human1 |  | GLS7 difference |  | -0.2807 |  |
|  |  | More readable is human1 |  | GLS6 difference |  | -0.0932 |  |
|  |  | More readable is human1 |  | GLS5 difference |  | -0.8651 |  |
|  |  | Confidence |  | Reaction time log |  | -0.1095 |  |
|  |  | Confidence |  | GLS Euclidean distance |  | -0.0626 |  |
|  |  | Confidence |  | GLS1 difference |  | 0.6796 |  |
|  |  | Confidence |  | GLS2 difference |  | -0.8108 |  |
|  |  | Confidence |  | GLS3 difference |  | -0.2743 |  |
|  |  | Confidence |  | GLS4 difference |  | 0.1229 |  |
|  |  | Confidence |  | id |  | 0.5153 |  |
|  |  | Confidence |  | GLS8 difference |  | 0.4863 |  |
|  |  | Confidence |  | GLS7 difference |  | 0.2745 |  |
|  |  | Confidence |  | GLS6 difference |  | -0.1273 |  |
|  |  | Confidence |  | GLS5 difference |  | 0.6892 |  |
|  |  | Reaction time log |  | GLS Euclidean distance |  | 0.7467 |  |
|  |  | Reaction time log |  | GLS1 difference |  | 0.0868 |  |
|  |  | Reaction time log |  | GLS2 difference |  | -0.0835 |  |
|  |  | Reaction time log |  | GLS3 difference |  | 0.4857 |  |
|  |  | Reaction time log |  | GLS4 difference |  | 0.5059 |  |
|  |  | Reaction time log |  | id |  | -0.0521 |  |
|  |  | Reaction time log |  | GLS8 difference |  | -0.4404 |  |
|  |  | Reaction time log |  | GLS7 difference |  | -0.6837 |  |
|  |  | Reaction time log |  | GLS6 difference |  | -0.7138 |  |
|  |  | Reaction time log |  | GLS5 difference |  | 0.1175 |  |
|  |  | GLS Euclidean distance |  | GLS1 difference |  | -0.0488 |  |
|  |  | GLS Euclidean distance |  | GLS2 difference |  | -0.0866 |  |
|  |  | GLS Euclidean distance |  | GLS3 difference |  | 0.6903 |  |
|  |  | GLS Euclidean distance |  | GLS4 difference |  | 0.7521 |  |
|  |  | GLS Euclidean distance |  | id |  | 0.1383 |  |
|  |  | GLS Euclidean distance |  | GLS8 difference |  | -0.1221 |  |
|  |  | GLS Euclidean distance |  | GLS7 difference |  | -0.4406 |  |
|  |  | GLS Euclidean distance |  | GLS6 difference |  | -0.6644 |  |
|  |  | GLS Euclidean distance |  | GLS5 difference |  | -0.0733 |  |
|  |  | GLS1 difference |  | GLS2 difference |  | -0.6408 |  |
|  |  | GLS1 difference |  | GLS3 difference |  | -0.5700 |  |
|  |  | GLS1 difference |  | GLS4 difference |  | 0.3898 |  |
|  |  | GLS1 difference |  | id |  | 0.1957 |  |
|  |  | GLS1 difference |  | GLS8 difference |  | 0.6171 |  |
|  |  | GLS1 difference |  | GLS7 difference |  | -0.2782 |  |
|  |  | GLS1 difference |  | GLS6 difference |  | 0.0360 |  |
|  |  | GLS1 difference |  | GLS5 difference |  | 0.3820 |  |
|  |  | GLS2 difference |  | GLS3 difference |  | -0.0440 |  |
|  |  | GLS2 difference |  | GLS4 difference |  | -0.3499 |  |
|  |  | GLS2 difference |  | id |  | -0.7701 |  |
|  |  | GLS2 difference |  | GLS8 difference |  | -0.5650 |  |
|  |  | GLS2 difference |  | GLS7 difference |  | 0.0128 |  |
|  |  | GLS2 difference |  | GLS6 difference |  | 0.5049 |  |
|  |  | GLS2 difference |  | GLS5 difference |  | -0.2486 |  |
|  |  | GLS3 difference |  | GLS4 difference |  | 0.3614 |  |
|  |  | GLS3 difference |  | id |  | 0.3990 |  |
|  |  | GLS3 difference |  | GLS8 difference |  | -0.3698 |  |
|  |  | GLS3 difference |  | GLS7 difference |  | -0.0685 |  |
|  |  | GLS3 difference |  | GLS6 difference |  | -0.7594 |  |
|  |  | GLS3 difference |  | GLS5 difference |  | -0.3239 |  |
|  |  | GLS4 difference |  | id |  | 0.4316 |  |
|  |  | GLS4 difference |  | GLS8 difference |  | 0.2738 |  |
|  |  | GLS4 difference |  | GLS7 difference |  | -0.3431 |  |
|  |  | GLS4 difference |  | GLS6 difference |  | -0.4114 |  |
|  |  | GLS4 difference |  | GLS5 difference |  | -0.1346 |  |
|  |  | id |  | GLS8 difference |  | 0.3300 |  |
|  |  | id |  | GLS7 difference |  | 0.3650 |  |
|  |  | id |  | GLS6 difference |  | -0.4468 |  |
|  |  | id |  | GLS5 difference |  | 0.0170 |  |
|  |  | GLS8 difference |  | GLS7 difference |  | -0.0369 |  |
|  |  | GLS8 difference |  | GLS6 difference |  | 0.1233 |  |
|  |  | GLS8 difference |  | GLS5 difference |  | -0.1925 |  |
|  |  | GLS7 difference |  | GLS6 difference |  | 0.4333 |  |
|  |  | GLS7 difference |  | GLS5 difference |  | 0.3075 |  |
|  |  | GLS6 difference |  | GLS5 difference |  | 0.0860 |  |
|  |  |  |  |  |  |  |  |
| --- | --- | --- | --- | --- | --- | --- | --- |
|  | | | | | | | |
